# Microscopic messengers: roles and prospects of extracellular vesicles in hepatitis B and C

**DOI:** 10.3389/fimmu.2026.1833204

**Published:** 2026-06-23

**Authors:** Lei Zhang, Ying Luo, Qingmei Ma, Yingtang Gao

**Affiliations:** 1 School of Medicine, Nankai University, Tianjin, China; 2 Department of Clinical Laboratory, Gansu Provincial Hospital, Lanzhou, China; 3 Tianjin Key Laboratory of Extracorporeal Life Support for Critical Diseases, Tianjin Institute of Hepatobiliary Disease, Nankai University Affiliated Third Center Hospital, Tianjin, China; 4 Central Hospital, Tianjin University, Tianjin, China; 5 Artificial Cell Engineering Technology Research Center, Tianjin, China

**Keywords:** biomarkers, dissemination, extracellular vesicles, hepatitis virus, immunomodulation, packaging, viral hepatitis

## Abstract

Extracellular vesicles (EVs) are important mediators of intercellular communication in hepatitis B virus (HBV) and hepatitis C virus (HCV) infection. EVs released from infected hepatocytes can carry viral nucleic acids, proteins, and regulatory non-coding RNAs to immune and nonimmune cells, thereby influencing viral dissemination, immune regulation, and disease progression. In particular, EV-associated cargos modulate antiviral immunity by affecting interferon signaling, natural killer cell function, cytokine production, immune checkpoint pathways, and T-cell exhaustion. These effects may promote viral persistence and immune evasion, although some EV populations can also enhance innate antiviral responses, indicating context-dependent dual roles. EVs also contribute to fibrosis and hepatocarcinogenesis by regulating hepatic stellate cell activation, inflammatory signaling, and tumor microenvironment remodeling. In addition, EV-derived RNAs and proteins show potential as noninvasive biomarkers and therapeutic targets. This review summarizes current evidence on EVs in HBV and HCV infection, with emphasis on immune regulation, viral persistence, disease progression, and translational prospects, while also discussing key challenges such as EV heterogeneity and co-isolation with viral particles.

## 1 Introduction

**Figure 1:**
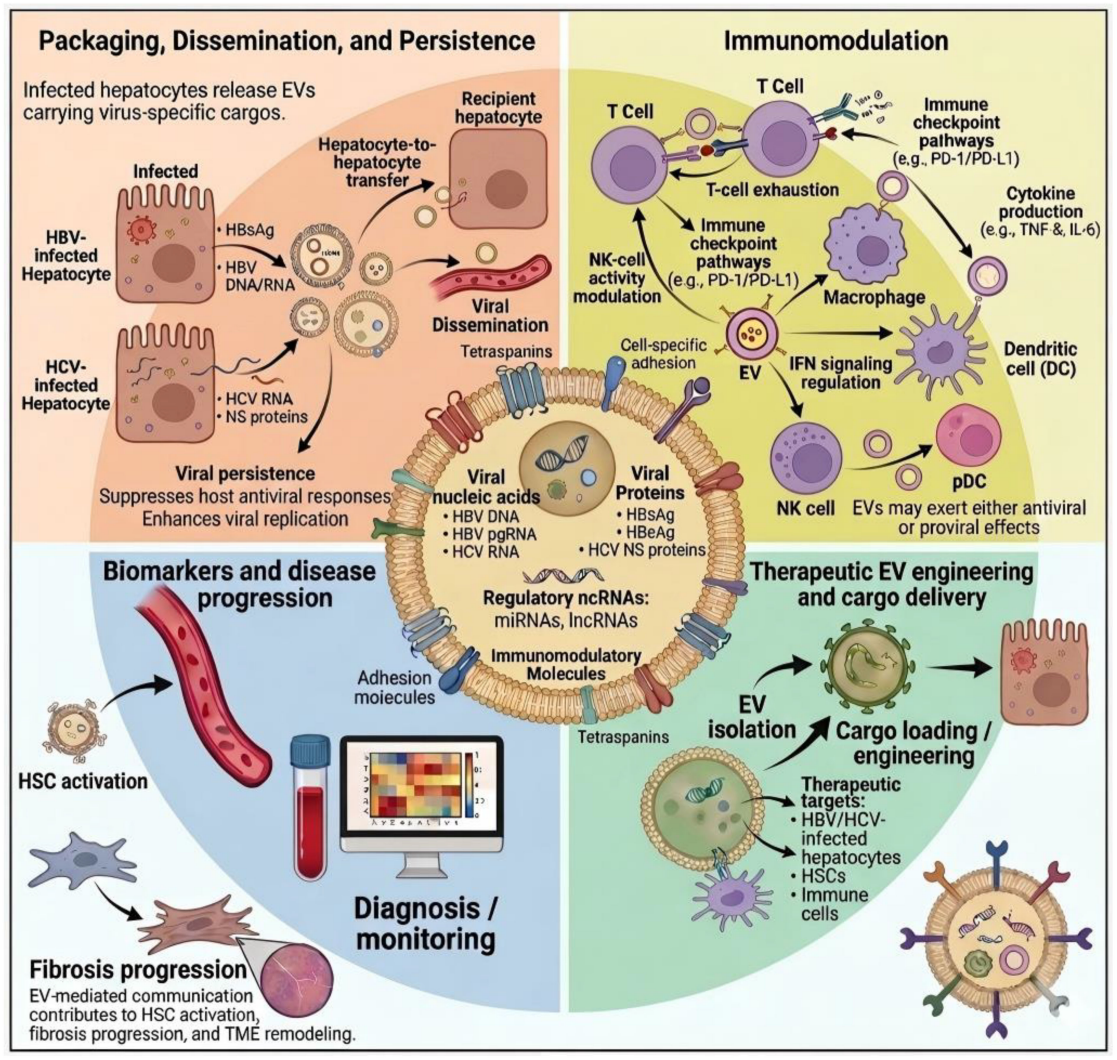
Integrated roles and mechanisms of extracellular vesicles in HBV and HCV infection. HBV- and HCV-infected hepatocytes release extracellular vesicles (EVs) carrying viral and host-derived cargos, including HBV DNA, HBV pgRNA, HCV RNA, viral proteins, regulatory noncoding RNAs, and immunomodulatory molecules. These EVs may mediate hepatocyte-to-hepatocyte cargo transfer, promote viral dissemination and persistence, and modulate antiviral immune responses. EVs also interact with T cells, NK cells, macrophages, dendritic cells, and plasmacytoid dendritic cells, thereby regulating interferon signaling, cytokine production, immune checkpoint pathways, and T-cell exhaustion. In addition, EV-mediated communication contributes to hepatic stellate cell activation, fibrosis progression, and tumor microenvironment remodeling. Circulating EV-associated RNAs and proteins may serve as noninvasive biomarkers for diagnosis and disease monitoring, while native or engineered EVs may be used as therapeutic carriers for antiviral or antifibrotic cargo delivery.

Viral hepatitis encompasses types A, B, C, D, E, and other variants. Hepatitis B and C pose significant global public health challenges due to their wide transmission routes. According to the WHO Global Hepatitis Report 2024, an estimated 254 million people were living with chronic HBV infection and approximately 50 million with chronic HCV infection in 2022. Viral hepatitis caused about 1.3 million deaths in 2022, mainly due to cirrhosis and hepatocellular carcinoma, highlighting that HBV and HCV remain major causes of liver-related mortality despite progress in vaccination, antiviral therapy, and HCV cure strategies (1). Hepatitis B virus (HBV) and hepatitis C virus (HCV) can be transmitted through blood contact, sexual intercourse, mother-to-child transmission, and sharing of contaminated syringes. These infections can lead to severe consequences such as chronic hepatitis, cirrhosis, and liver cancer (1). The liver is a vital organ that plays a crucial role in maintaining body homeostasis. Persistent or severe infections may result in chronic liver disease, cirrhosis development, and even the onset of liver cancer (2). understanding non-classical mechanisms of viral persistence, immune escape, and liver disease progression is essential. EVs are increasingly recognized as such mechanisms because they can transport viral nucleic acids, proteins, lipids, and regulatory RNAs between infected hepatocytes, immune cells, and stromal cells, thereby linking viral replication with inflammation, fibrosis, and carcinogenesis. In recent years, there has been growing interest in the involvement of extracellular vesicles (EVs) in viral hepatitis pathogenesis. These microscopic messengers including exosomes, microvesicles, and apoptotic bodies have emerged as key players influencing the progression of hepatitis viruses like HBV, HCV, and other infectious agents affecting the liver (3). This review aims to provide an enhanced understanding of the multifaceted roles played by EVs in hepatitis B and C while exploring their potential as therapeutic targets. The integrated roles of EVs in viral dissemination, immune regulation, disease progression, biomarker development, and therapeutic delivery are summarized in **Figure 1**.

## 2 EVs: vehicles of microscopic messengers

EVs are nanoscale vesicles enclosed by a lipid bilayer that can be secreted by nearly all cell types (4). EVs can be categorized based on their biogenesis process (exosomes, microvesicles, and apoptotic bodies) or size (small EVs < 200 nm and medium/large EVs ≥ 200 nm) (5). Exosomes, microvesicles, and apoptotic bodies differ in their biogenesis pathways, size distribution, and biological characteristics. Exosomes are generally small vesicles (approximately 30–150 nm) generated through the endosomal pathway and released after fusion of multivesicular bodies with the plasma membrane. In contrast, microvesicles are typically larger vesicles (approximately 100–1000 nm) formed by direct outward budding and shedding of the plasma membrane. Apoptotic bodies are usually the largest and most heterogeneous EV subtype, released during programmed cell death and containing cellular organelles, nuclear fragments, and cytoplasmic components. However, because EV subtypes often overlap in size, composition, and isolation properties, the updated MISEV guidelines recommend using operational terms such as small EVs and medium/large EVs when biogenesis pathways are not directly demonstrated experimentally. It should be noted that EV nomenclature and characterization remain important methodological issues. The updated MISEV2023 recommendations emphasize that EV studies should report the source of EVs, pre-analytical variables, separation methods, particle characterization, and functional validation to improve reproducibility and transparency (6). In viral hepatitis research, this issue is particularly important because EVs, lipoproteins, subviral particles, and complete virions may overlap in size and density. Therefore, the term “EVs” should be used cautiously unless vesicle preparations are validated by multiple complementary methods, such as nanoparticle tracking analysis, transmission electron microscopy, density-gradient separation, and detection of EV-enriched proteins including CD9, CD63, CD81, ALIX, and TSG101.

Mechanistically, EV biogenesis and release are regulated by multiple membrane-trafficking pathways (7–9). Exosome formation involves inward budding of endosomal membranes and the generation of multivesicular bodies, which can be mediated by ESCRT-associated proteins such as ALIX and TSG101, as well as ESCRT-independent mechanisms involving ceramide and tetraspanin-enriched membrane domains (7, 8). Mature multivesicular bodies subsequently fuse with the plasma membrane through Rab GTPase- and SNARE-associated pathways, leading to exosome release (9). In contrast, microvesicles are generated by outward budding of the plasma membrane through cytoskeletal remodeling, phospholipid redistribution, and actomyosin contraction (8). After release, EVs attach to and enter recipient cells through receptor-ligand interactions, membrane fusion, endocytosis, or macropinocytosis (9). Surface molecules such as integrins, tetraspanins including CD9, CD63, and CD81, phosphatidylserine-binding receptors, and heparan sulfate proteoglycans contribute to EV recognition, uptake, and tissue tropism (8, 9). These processes are particularly relevant in HBV and HCV infection, as hepatotropic viruses may exploit EV biogenesis and uptake pathways to promote viral dissemination, immune modulation, and persistence (10).

Initially underestimated as carriers for cellular waste disposal, EVs are now recognized as pivotal mediators in intercellular communication (7, 11). The mechanisms through which EVs transmit information include: ①Releasing EVs into the intercellular space or tissue microenvironment and reaching target cells via systemic or local circulation; ②Activation of recipient cell receptors by ligands present on the surface of EVs to facilitate communication; ③Specific receptor cells absorbing EVs and releasing their cargo into the cytoplasm (12), Once released, EVs mediate cell-cell interactions through transfer to enable a wide range of physiological and pathological processes. Encapsulated signaling factors within EVs remain protected from enzymatic activity and can be transported to distant organs or tissues to facilitate interorgan crosstalk (13).

As a crucial mediator of intercellular communication, EVs play an essential role in maintaining cell homeostasis and physiological processes while also contributing to the development of diseases. Research has demonstrated that EVs are involved in all aspects of hepatitis virus packaging, transmission, immune regulation, antiviral response, and more. Through intracellular material secretion and intercellular material and information exchange within the local microenvironment, EVs significantly contribute to the pathogenesis of viral hepatitis (14–16). The diagnostic potential of EVs in chronic liver disease lies in their varying contents that change during different stages of the disease progression, allowing for monitoring purposes (2, 17). Furthermore, unmodified or engineered EVs have garnered attention as novel therapeutic agents or carriers.

In terms of liver sources for EVs production, they can be secreted by various cells within the liver itself or from other tissues/organs that enter through blood circulation. Most cell types found within the liver have been shown to produce EVs under *in vitro* conditions such as hepatocytes, cholangiocytes hepatic stellate cells (HSC), sinusoidal endothelial cells (SEC), Kupffer cells along with other diverse immune cell populations (18). These EVs may cross endothelial barriers via transcellular or paracellular transport pathways; however paracellular transport is particularly important when it comes to transporting these vesicles through gaps between endothelial cells (18).

HBV and HCV infection are major causes of chronic liver disease worldwide and remain leading contributors to liver-related mortality despite advances in antiviral therapy and vaccination strategies. According to recent WHO estimates, hundreds of millions of individuals worldwide are chronically infected with HBV or HCV, and a substantial proportion of infected patients eventually develop progressive liver diseases, including fibrosis, cirrhosis, hepatic decompensation, and hepatocellular carcinoma (HCC). Chronic HBV infection is characterized by the persistence of covalently closed circular DNA (cccDNA) within hepatocytes, whereas HCV persistence is associated with continuous RNA replication and high viral genetic variability. These features allow both viruses to evade complete immune clearance and establish long-term infection.

Importantly, liver injury in chronic viral hepatitis is not caused solely by direct viral cytopathic effects, but largely results from persistent immune-mediated inflammation and dysregulated antiviral responses. During chronic infection, continuous activation of innate and adaptive immune pathways promotes hepatocyte stress, inflammatory cytokine release, oxidative damage, and recruitment of immune cells into the hepatic microenvironment. Prolonged inflammatory signaling subsequently activates hepatic stellate cells (HSCs), leading to excessive extracellular matrix deposition and progressive fibrosis. Over time, chronic inflammation, fibrosis-associated tissue remodeling, angiogenesis, and immune exhaustion collectively create a pro-tumorigenic microenvironment that facilitates the development of HCC.

## 3 EV biogenesis and viral hijacking pathways

HBV and HCV can exploit these vesicle biogenesis pathways for different biological purposes. Recent evidence further supports the concept that hepatotropic viruses actively hijack host EV biogenesis machinery to facilitate viral persistence and immune modulation. HBV and HCV can exploit ESCRT-associated pathways, tetraspanin-enriched membrane domains, and multivesicular body trafficking to promote the packaging and dissemination of viral components through EV-associated routes. Importantly, the overlap between EV secretion pathways and viral assembly mechanisms complicates the distinction between authentic EV cargo and co-isolated virions, representing a major methodological challenge in the field (10).

HBV assembly and release are associated with endosomal sorting pathways and tetraspanin-enriched compartments, while HCV replication and spread are closely linked to intracellular membrane rearrangements and lipid-associated vesicular trafficking. This overlap between viral assembly pathways and EV biogenesis provides a mechanistic basis for viral components entering EVs. As a result, infected hepatocytes may release vesicles containing viral DNA, viral RNA, viral proteins, host miRNAs, and immune-regulatory molecules, allowing viruses to influence neighboring uninfected hepatocytes and immune cells without relying exclusively on free virions.

## 4 EVs in the packaging of hepatitis viruses

Studies have reported the presence of various HBV-related components in EVs derived from HBV-infected hepatocytes, including HBV DNA, RNA, HBsAg, and HBeAg. This suggests the potential of inducing active infection in human liver cells (18). EVs can be isolated from the supernatant of HBV-infected liver cells, characterized by the presence of both EVs markers and HBV-specific components. Electron microscopy has confirmed the presence of intact viral particles within EVs, and the surface of EVs derived from HBV-expressing cells has been found to carry hepatitis B virus surface antigen (3). The study by Ninomiya et al. indicates that the exosome-associated tetraspanin CD63 contributes to the efficient assembly and infection of hepatitis B virus (19), The findings suggest a potential shared packaging mechanism between exosomes and HBV Dane particles. The exosome-associated tetraspanin CD63 plays a role in the effective assembly and dissemination of HBV (20). HBV is a partially double-stranded DNA virus that establishes persistence via covalently closed circular DNA (cccDNA) and exploits the MVB/ESCRT pathway for virion assembly.

Masciopinto et al. co-transfected mammalian cells with cDNA encoding the HCV envelope proteins E1-E2 and human tetraspanin CD81 (huCD81). They confirmed that huCD81 facilitates the assembly, maturation, and transportation of HCV envelope proteins in the Golgi apparatus. Furthermore, these proteins are secreted in the form of exosomes, with the secreted 60-100-nm membrane vesicles (exosomes) being rich in CD81 and possessing fusogenic activity (21). By contrast, HCV is a positive-sense single-stranded RNA virus whose replication occurs on endoplasmic reticulum-derived membranous webs, and its EV-mediated spread depends heavily on the Ago2-miR122-HSP90 complex. Dreux et al.’s study demonstrates that, following hepatitis C virus (HCV) infection of liver cells, HCV RNA is released in the form of exosomes, sufficient to activate plasmacytoid dendritic cells (pDCs). The exosomal transfer of viral RNA depends on the endosomal sorting complex required for transport (ESCRT) machinery and membrane-associated protein A2 (Annexin A2, a RNA-binding protein involved in membrane vesicle transport). Moreover, this process can be inhibited by exosome release inhibitors, ultimately resulting in exosomes containing HCV RNA (22). Subsequently, Ramakrishnaiah et al, confirmed through RT-PCR, mass spectrometry, and transmission electron microscopy analysis that purified EVs isolated from HCV-infected human liver cancer Huh7.5.1 cells contain full-length viral RNA, viral proteins, and particles. Furthermore, these EVs were capable of transferring HCV to uninfected human liver cancer Huh7.5.1 cells, leading to persistent infection (23). Liu et al. also confirmed the presence of HCV RNA, E2 protein, and core protein in EVs. The detection rate of EVs associated HCV RNA in the plasma of HCV-infected patients was higher than that of non-EVs-associated free HCV RNA. This suggests that HCV RNA primarily exists in the form of EVs-associated HCV RNA in the plasma of HCV patients, indicating the significant research value of the non-receptor-dependent mode of HCV infection (24).

## 5 EVs in the dissemination of hepatitis viruses

Various processes in liver diseases, such as activation of the hepatic innate immune system, activation of hepatic stellate cells, hepatocyte apoptosis, organelle dysfunction, and systemic inflammation, may lead to the release of EVs (25). Research has demonstrated the isolation of EVs from the serum of immune-tolerant (IT) chronic hepatitis B (CHB) patients. These EVs contain HBV viral components and can induce active infection in uninfected human liver cells. This suggests that HBV can utilize EVs for intercellular dissemination (26). EVs can associate with proteins related to the endosomal sorting complex required for transport (ESCRT) and undergo transport through a ceramide-dependent pathway, thereby achieving the dissemination of the virus (27). Therefore, EVs can efficiently propagate HBV by providing a biological mechanism, serving as an immune barrier, and enhancing HBV replication capability. In an animal model of tree shrews infected with HBV, Kouwaki et al. discovered that HBV-infected hepatocytes could release EVs containing viral nucleic acids through MyD88, TICAM-1, and MAVS-dependent pathways (15).

Masciopinto et al. not only confirmed the synergistic interaction between the HCV envelope proteins and the human tetraspanin CD81 using cell models but also identified HCV RNA in EVs from the plasma of HCV-infected patients. The study suggests that even in the presence of neutralizing antibodies, the HCV/CD81 complexes exit cells in the form of exosomes, participate in circulation, and utilize the fusion capability of these vesicles to infect new cells (21). HCV-infected hepatocytes can release EVs carrying HCV RNA and proteins, delivering their contents to other liver cells. Due to the protective role of EVs, this mode of dissemination can, to some extent, diminish the effectiveness of antiviral antibodies. These findings also support the emerging concept that the traditional distinction between enveloped and nonenveloped viruses may be overly simplistic. Increasing evidence suggests that certain hepatotropic viruses can exploit host-derived membrane structures and EV-associated pathways to acquire “quasi-enveloped” characteristics, thereby facilitating immune evasion, extracellular stability, and noncanonical transmission routes (28). In this context, EV-associated viral dissemination represents not merely passive cargo transport but a sophisticated virus-host adaptation strategy that blurs the classical boundary between viral particles and host membrane vesicles.

This mechanism may offer an effective pathway for HCV immune evasion (19, 29). By isolating EVs from individuals infected with HCV, The Ago2-miR122-HSP90 complex within EVs ensures the replicative capability of HCV RNA and its subsequent infection and spread by binding to the 5’ untranslated region (5UTR) of the HCV. Furthermore, it was observed that EVs loaded with miR-122 inhibitors effectively suppressed HCV transmission mediated by EVs in hepatocytes (14), This indicates that targeting EVs-derived miRNA can also inhibit the spread of HCV.

The expression of long non-coding RNAs (lncRNAs) has been found to facilitate the spread of HCV infection. For instance, it has been reported that the expression of long non-coding RNA, Nuclear Enriched Abundant Transcript 1 (NEAT1), and Taurine Upregulated Gene 1 (TUG1) is decreased in the serum of both HCV patients and HCV-infected hepatocellular carcinoma (HCC) patients. The detectability of serum lncRNAs and their diagnostic significance have been highlighted (30). Beyond individual studies, it is important to distinguish between mechanistic evidence—obtained from well-defined cell or animal models with functional readouts—and observational associations. The following lncRNA findings represent largely correlative observations. The expression of the lncRNA Insulin-Like Growth Factor 2 (IGF2) (31), lncRNA HOTAIR (32)and lncRNA-hulc (33)will upregulated upon viral invasion of host cells. The upregulation of these lncRNAs promotes the spread of viral infection by assisting in viral replication and release. Zhang et al. research team found that the expression of lncRNA-HEIH is elevated in the EVs and serum of HCV-infected HCC patients (34).

EVs in HCV also provide a feedback loop that can exacerbate or alleviate infection, inflammation, and endothelial dysfunction in the body (35, 36). This dual role of EVs in infectious hepatitis makes them a “double-edged sword”. On one hand, they can promote viral infection by transporting the virus to other uninfected cells, providing a “shield” for viral particles and RNA during antibody-mediated immune responses. On the other hand, EVs containing antiviral substances or originating from immune cells can inhibit viral infection. Thus, EV delivery may represent an effective cellular adaptive mechanism to enhance cell survival under viral stress or to support the survival of the virus itself during hepatitis infection. Although both HBV and HCV can use EVs to enhance viral persistence, their EV-mediated mechanisms are not identical. HBV is a DNA virus that maintains persistence through cccDNA and produces abundant viral antigens and subviral particles. Therefore, HBV-related EVs may mainly contribute to antigen transport, immune modulation, and intercellular transfer of HBV DNA/RNA. In contrast, HCV is an RNA virus that depends on host lipid metabolism, miR-122, and membranous replication complexes. HCV-associated EVs can transfer replication-competent RNA and host factors such as Ago2, miR-122, and HSP90, thereby supporting receptor-independent infection and partial escape from neutralizing antibodies. This difference suggests that EV-targeted strategies in HBV may need to focus more on antigen presentation, immune exhaustion, and cccDNA-related persistence, whereas EV-targeted strategies in HCV may focus more on RNA transfer, miR-122-dependent replication, and post-SVR liver disease progression (**Table 1**).

**Table 1:** Comparison of EV-mediated mechanisms in HBV and HCV infection.

Feature	HBV	HCV
Viral genome	Partially double-stranded DNA	Positive-sense single-stranded RNA
Major persistence mechanism	cccDNA persistence, antigen production	RNA replication complex, lipid-associated spread
EV-associated viral cargo	HBV DNA, HBV RNA, HBsAg, HBeAg, HBcAg	HCV RNA, core protein, E2 protein
Key EV-related molecules	CD63, CD81, ESCRT components, ceramide pathway	CD81, Ago2, miR-122, HSP90
Main biological effect	Viral dissemination, immune suppression, NK/T-cell dysfunction	Receptor-independent spread, antibody escape, persistent replication
Immune modulation	IL-12/IL-21 inhibition, PD-L1 induction, NK dysfunction	Galectin-9 induction, MDSC expansion, Tfh/Tfr imbalance
Biomarker candidates	HBV-miR-3, miR-122, miR-192, miR-22	miR-155, miR-122, lncRNA-HEIH, lnc-DANCR
Therapeutic implication	Block EV-mediated immune suppression and viral cargo transfer	Target EV-associated HCV RNA transfer and miR-122-dependent replication

**Figure 2:**
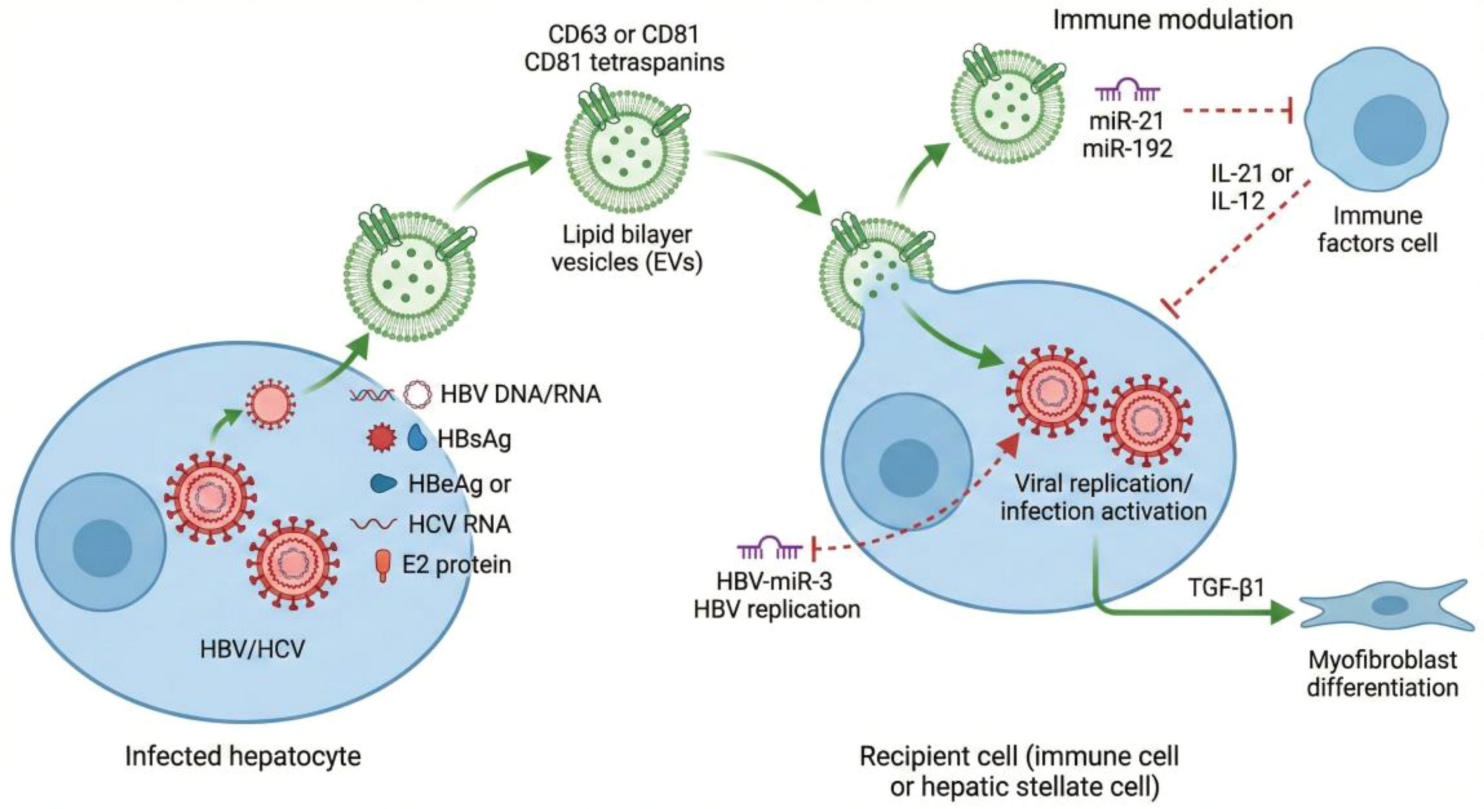
Extracellular vesicle-mediated transfer of viral and regulatory cargos during HBV/HCV infection. HBV/HCV-infected hepatocytes release extracellular vesicles (EVs) containing viral and host-derived cargos, including HBV DNA/RNA, HCV RNA, viral proteins, and regulatory microRNAs. These EVs, characterized by lipid bilayers and tetraspanins such as CD63 and CD81, can be taken up by recipient cells, including immune cells and hepatic stellate cells. EV-mediated cargo transfer may promote viral replication and infection activation in recipient cells, modulate antiviral immunity by regulating factors such as IL-12 and IL-21, and contribute to fibrosis-related responses such as myofibroblast differentiation through mediators including TGF-β1. Meanwhile, specific EV-associated microRNAs, such as miR-21, miR-192, and HBV-miR-3, may exert either proviral or antiviral effects depending on their targets and cellular context. This figure illustrates the dual role of EVs in viral dissemination and immune regulation during hepatitis virus infection.

## 6 Immunoregulation

The immunomodulatory roles of EVs in viral hepatitis are multifaceted and can be organized into three functional domains: regulation of innate immunity, modulation of adaptive immunity, and immune evasion mechanisms.

EV-mediated immune regulation in viral hepatitis should be understood as a bidirectional process. On one hand, EVs released from infected hepatocytes can deliver viral nucleic acids to macrophages, dendritic cells, and plasmacytoid dendritic cells, activating pattern-recognition receptor pathways and inducing interferon-related antiviral responses. On the other hand, the same or similar EV populations may carry host miRNAs, viral miRNAs, checkpoint-related molecules, or immunosuppressive proteins that inhibit NK-cell activity, weaken dendritic-cell function, and promote T-cell exhaustion. Thus, EVs do not act simply as proviral or antiviral mediators; their biological effect depends on their cellular origin, cargo composition, target cell type, and stage of infection (15).

The following sections describe how EVs carrying specific molecular cargo sequentially shape these immunological processes during HBV and HCV infection.

### 6.1 EV-mediated regulation of innate immunity

Interleukin-21 (IL-21) is secreted by activated CD4+ T cells and acts on various immune cells to regulate diverse immune responses. Enomoto et al, through the analysis of EVs released by HBV-infected human liver cells, identified that these EVs carry miR-21, miR-192, miR-215, miR-221, and miR-222. These miRNAs directly target multiple sequences in the 3’UTR of human IL-21 mRNA. They can inhibit the expression of IL-21 in human T cells, ultimately impairing the antiviral capacity of the human body (**Table 2**) (15, 37). IL-12 is a well-known cytokine that activates NK cells. HBV-infected liver cells can reduce the activation of NK cells by regulation the expression of IL-12 through the release of EVs loaded with miR-21. This may be a crucial mechanism by which the virus modulates the host’s innate immune response (15). Furthermore, the study also identified that HBV-miR-3 has inhibitory effects on HBsAg, HBeAg, and HBV replication (2). HBV-miR-3 is highly expressed in peripheral blood EVs of patients with hepatitis B, and it exhibits inhibitory effects on HBV replication by targeting the 3.5 kb HBV transcript (16). miR-3 also possesses antiviral immune effects. Studies indicate that HBV-miR-3, by downregulating the expression of SocS5 in hepatocytes, activates the JAK/STAT signaling pathway, enhances interferon (IFN) induced antiviral effects against HBV, and promotes M1 polarization of macrophages and the secretion of IL-6 (38). Hepatitis B surface antibody (HBsAb) is a crucial immune protein that provides resistance against HBV infection. The protective effect of HBsAb is significantly reduced in the presence of HBcAg+ CD81+ EVs, suggesting that CD81+ EVs are involved in the immune evasion of HBV (39). Kouwaki et al. utilized a tree shrew model infected with HBV and found that EVs containing viral nucleic acid can stimulate the expression of NKG2D ligands in macrophages (15). The expression of NKG2D ligands in macrophages activates NK cells, and early during viral infection, NK cells produce IFNγ, promoting the degradation of HBV nucleic acids in the cytoplasm, thereby exerting antiviral effects, On the other hand, studies suggest that the uptake of HBV-positive EVs by NK cells impairs NK cell functions, including IFN production, cytotoxic activity, NK cell proliferation and survival, as well as the response to poly I:C stimulation (26).

**Table 2:** Functions of EVs miRNAs in HBV/HCV-infected cells.

microRNA	Virus	Expression	Functions	Reference
miR-3	HBV	Up	Inhibition of the replication of HBsAg, HBeAg, and HBV	(2)
miR-21	HBV	Up	Suppressing the expression of IL-12 and IL-21 inhibits immune responses	(15, 37)
miR-19a	HCV	Up	Modulating the SOCS-STAT3 axis activates hepatic stellate cells (HSCs), promoting liver fibrosis	(37)
miR-124	HCV	Down	Downregulation promotes MDSC expansion and immunosuppressive signaling	(48)
miR-192	HBV /HCV	Up	Inhibiting the expression of IL-21 suppresses immune responses and promotes liver fibrosis	(37, 45)
miR-215	HBV	Up	Suppressing the expression of IL-21 inhibits immune responses	(37)
miR-221	HBV	Up	Suppressing the expression of IL-21 inhibits immune responses	(37)

### 6.2 EV-mediated modulation of adaptive immunity

Negative regulatory factors in the IFN signaling pathway inhibit the immune response in the liver, leading to suboptimal treatment responses to IFNα in patients with CHB. Therefore, elucidating key negative factors of IFNα and clarifying their regulatory mechanisms are crucial for improving the efficacy of IFNα in anti-HBV therapy. Shi et al. discovered that EV-mediated transport of interferon-induced transmembrane protein 2 (IFITM2) to dendritic cells (DCs) suppressed the activation of the IFNα pathway and blocked the antiviral effects of exogenous IFNα against HBV (40). EVs secreted by HBV infected cells are phagocytosed by monocytes, and monocytes exert immune inhibitory effects by upregulating PD-L1 expression and inhibiting CD69 expression (41). PD-1 (Programmed Death-1) under physiological conditions maintains the homeostasis of T cells by inhibiting T cell activation and proliferation. In patients with CHB, T cell depletion and inactivation are observed, possibly influenced by EVs associated with HBV, leading to an increased expression of PD-L1 (Programmed Death Ligand-1) in monocytes (42, 43).

### 6.3 Antiviral EV cargo and immune evasion in HCV

Studies indicate that EVs isolated from the serum of HCV liver fibrosis patients contain higher levels of miR-19a compared to EVs obtained from healthy volunteers and non-HCV-related liver disease patients. MiR-19a regulates the SOCS-STAT3 axis to activate HSC. Therefore, it is inferred that EV-mediated intercellular communication plays a significant role in HSC activation leading to liver fibrosis after HCV infection (44). Similarly, Kim et al. observed upregulation of miR-192 expression in HCV-infected liver cells. MiR-192 can be transmitted to HSC through EVs, stimulating the differentiation of HSC into myofibroblasts through the activation of TGF-β1 (**Table 1**) (45). Galectin-9 (gal-9) is a natural ligand for T cell immunoglobulin and mucin domain protein 3 (Tim-3), with Its high expression increasing the number of regulatory T cells and accelerating the apoptosis of HCV-specific cytotoxic T lymphocytes (CTL), this contributes to the maintenance of chronic HCV infection (46). The research by Harwood et al. indicates that a portion of HCV RNA stimulates the production of gal-9 in monocytes through EVs, thereby contributing to chronic HCV infection. Both subgenomic replicon cells (SGR) and EVs from HCV-infected cells can induce the production of gal-9 in monocytes. Furthermore, the differentiation of monocytes is associated with an increase in gal-9 levels. In comparison to normal controls, HCV patients exhibit elevated levels of gal-9 in monocytes (47). HCV-associated EVs have the capability to regulate the expression of Homeobox A Transcriptional Interference RNA Marrow Specificity 1 (HOTAIRM1), an antisense RNA, and its target gene HOXA1 in myeloid-derived suppressor cells (MDSCs). Subsequently, this process stimulates the production of immune inhibitory factors by modulating the expression of miR-124, thereby promoting the sustained infection of HCV (**Table 1**) (48). Reports indicate that plasma EVs from HCV-infected patients and supernatant from HCV-infected liver cells can transfer HCV RNA to myeloid-derived suppressor cells (MDSCs). This transfer, accompanied by the downregulation of miRNA-124 expression, promotes the expansion of MDSCs. Subsequently, the expansion of MDSCs facilitates the differentiation of T follicular regulatory cells (Tfr), increases the production of interleukin IL-10, inhibits the function of T follicular helper cells (Tfh), and suppresses the secretion of IFN-γ. These events collectively result in sustained viral infection, outlining a novel mechanism by which EVs modulate the immune response during HCV infection (49). The EV-mediated transfer of viral and regulatory cargos between infected hepatocytes and recipient cells is schematically illustrated in **Figure 2**.

Taken together, EVs orchestrate a coordinated immunosuppressive program in chronic HBV and HCV infection: EVs carrying miR-21, miR-192, and IFITM2 sequentially dampen innate sensing (NK cell activation, IFN-α signaling), while EVs promoting PD-L1 upregulation on monocytes and galectin-9 production act in concert to exhaust virus-specific T cells, ultimately creating an immunosuppressive microenvironment that favors viral persistence.

In summary, the role of EVs in infectious hepatitis varies based on their cargo. When EVs carry viral proteins, nucleic acids, and other substances, they facilitate the spread of HBV and HCV. Conversely, when EVs contain anti-HBV and anti-HCV factors, they exhibit inhibitory effects on viral activity and play a role in antiviral defense. Furthermore, the substances carried by EVs can serve as ligands for immune cells, activating antiviral immune responses and exerting a positive influence. The association between EVs and the transmission of hepatitis viruses represents a rapidly evolving research field, playing a crucial role in influencing immune responses.

Collectively, these findings support an integrated model in which EVs function as central coordinators of immune remodeling during HBV and HCV infection. EVs released from infected hepatocytes carry distinct viral and host-derived cargos, including viral nucleic acids, miRNAs, lncRNAs, and immunomodulatory proteins, which sequentially target multiple immune pathways and cell populations. At the innate immune level, EV-associated miR-21 and related regulatory miRNAs suppress IL-12 and IL-21 signaling, thereby impairing NK-cell activation and antiviral cytokine production. Simultaneously, EV-mediated delivery of IFITM2 and other inhibitory factors attenuates interferon signaling in dendritic cells and weakens antiviral immune priming. At the adaptive immune level, EVs contribute to T-cell exhaustion and immune tolerance through induction of PD-L1 expression, galectin-9/Tim-3 signaling, and expansion of immunosuppressive myeloid-derived suppressor cells (MDSCs). These pathways collectively suppress cytotoxic T-cell responses, alter Tfh/Tfr balance, and enhance IL-10-mediated immunosuppressive signaling, ultimately favoring chronic viral persistence. Importantly, EV-mediated immune regulation is highly context-dependent and may exert both antiviral and proviral effects depending on EV origin, cargo composition, recipient cell type, and infection stage. Therefore, EVs should not be regarded merely as passive carriers of viral material, but rather as dynamic immunological regulators that integrate innate immune sensing, immune checkpoint signaling, inflammatory responses, and fibrosis-associated immune remodeling during HBV and HCV infection. Research in this area provides valuable insights into understanding the immunological mechanisms of viral infections and developing new therapeutic strategies.

## 7 EVs in the progression from chronic hepatitis to fibrosis and hepatocellular carcinoma

Chronic HBV and HCV infection can gradually progress from persistent hepatic inflammation to fibrosis, cirrhosis, and hepatocellular carcinoma. EVs may participate in this disease continuum by connecting injured hepatocytes, hepatic stellate cells, endothelial cells, macrophages, and tumor cells. During chronic infection, hepatocyte-derived EVs can transfer inflammatory signals and non-coding RNAs to hepatic stellate cells, promoting their activation into collagen-producing myofibroblast-like cells. This process is closely associated with profibrotic pathways such as TGF-β, STAT3, NF-κB, and PI3K/AKT signaling.

In HCV infection, EV-associated miR-19a and miR-192 have been shown to activate hepatic stellate cells and promote fibrogenic transformation. In addition, EVs released under persistent inflammatory conditions may reshape the liver microenvironment by promoting macrophage polarization, endothelial dysfunction, angiogenesis, and extracellular matrix remodeling. These changes provide a favorable niche for cirrhosis and hepatocarcinogenesis.

EVs may also contribute to viral hepatitis-associated HCC by transferring oncogenic RNAs and proteins. HCC-derived EVs can be taken up by hepatocytes or surrounding stromal cells and alter the expression of genes involved in proliferation, migration, immune escape, and tumor microenvironment remodeling. Recent evidence suggests that EV-derived mRNAs and non-coding RNAs may not only reflect tumor burden but also actively participate in tumor progression, supporting their dual role as pathogenic mediators and early diagnostic biomarkers (50). Therefore, EVs should be viewed as biological bridges linking viral persistence, chronic inflammation, fibrogenesis, and malignant transformation.

## 8 Application of EVs as biomarkers

EV-based biomarkers have several theoretical advantages over conventional soluble biomarkers. First, the lipid bilayer of EVs protects RNA and protein cargo from enzymatic degradation, improving molecular stability in blood and other body fluids. Second, EV cargo partly reflects the physiological or pathological state of parental cells, allowing circulating EVs to provide information about injured hepatocytes, activated stellate cells, immune cells, and tumor cells. Third, EV-associated nucleic acids may improve the sensitivity of liquid biopsy, especially in patients with early-stage liver injury or low alpha-fetoprotein HCC. However, most reported EV biomarkers in HBV- and HCV-related diseases still require multicenter validation, standardized isolation protocols, and comparison with established clinical markers such as HBV DNA, HBsAg, HBeAg, ALT, AFP, APRI, FIB-4, and liver stiffness measurement.

Existing research indicates that peripheral blood-derived EVs can offer non-invasive diagnostics, serving as a potential tool for early disease detection (51), They can also serve as candidate biomarkers for predicting the progression of HBV-related liver diseases (52). The constructed plasma-derived EVs small non-coding RNA (sncRNA) profile can serve as a reliable biomarker for detecting HBV-related liver injury. It can also function as a predictive marker for selecting adjunctive therapies, in patients with HBV infection, HBV-miR-3 is released into circulation through both EVs and HBV viral particles. In the serum of patients during the acute phase of HBV infection, the expression of HBV-miR-3 is positively correlated with HBV titers (52). Levels of miR-192-5p, miR-193b-3p, miR-194-5p, miR-122, and miR-22 derived from EVs are significantly elevated in HBeAg-positive patients and are correlated with HBV DNA levels and HBsAg titers (53, 54). miR-122 is considered a biomarker for various liver injuries, and its elevated expression is associated with HBV infection. Serum levels of miR-122 in patients with CHB are significantly higher than those in healthy individuals. Therefore, miR-122 can reflect the degree of liver inflammation and is associated with the progression of HBV infection (53).

EVs can serve as early and non-invasive diagnostic biomarkers for HCV-related HCC. In a study conducted in 2020 by Ghosh and colleagues, a combination of seven EVs miRNAs, including 10b-5p, 221-3p, 223-3p, 10b-5p, 221-3p, 223-3p, and 21–5 was identified as sensitive markers for early diagnosis of HCC, regardless of the HCV etiology. This is particularly relevant for HCC associated with low alpha-fetoprotein levels (55). Wang et al. utilized EVs isolated from tissues and serum of HCV-infected HCC patients following curative liver resection. They proposed that the expression of long non-coding RNA (lncRNA) named lnc-DANCR in EVs is positively correlated with the recurrence of HCV-related HCC. Consequently, the copy number of lnc-DANCR is employed as a biomarker to predict the recurrence and mortality rates of HCV-related HCC (56). Additional studies have reported that the expression of lncRNA-HEIH is highest in EVs extracted from the serum of HCV-related HCC patients, followed by HCV-induced cirrhotic EVs, and is lowest in EVs from individuals with chronic HCV infection. Both serum and EVs-associated lncRNA-HEIH can be utilized to assess the potential of HCV-related HCC (34). Matboli et al. reported that the expression of lncRNA-RP11-583F2.2 in EVs from the serum of HCC patients is higher compared to both HCV patients and healthy individuals. This finding suggests that EVs lncRNA-RP11-583F2.2 may serve as a diagnostic and prognostic biomarker for HCC. However, whether EVs lncRNA-RP11-583F2.2 can function as a biomarker for HCV-related HCC and a regulatory factor in HCV infection remains to be elucidated (57).

It is important to distinguish between clinically validated EV-associated biomarkers and exploratory molecular findings. Certain EV-associated miRNAs, such as miR-122 and miR-192, have been repeatedly associated with liver injury, fibrosis progression, and viral hepatitis severity across multiple clinical studies, supporting their potential translational relevance. In contrast, several recently identified EV-associated lncRNAs and miRNAs, including lncRNA-HEIH, lnc-DANCR, and other candidate non-coding RNAs, remain exploratory biomarkers that are primarily supported by limited cohort studies or experimental observations. Although these molecules may provide important mechanistic insights into viral persistence, immune regulation, or hepatocarcinogenesis, their diagnostic specificity, reproducibility, and clinical utility require further validation in large multicenter studies before routine clinical application.

Significant variations in treatment responses are observed among patients infected with different HCV genotypes. Patients infected with genotype 1b are predominantly associated with sustained virological response (SVR), while those with genotype 6a are mainly associated with non-response (NR). The expression levels of EVs miRNA-155 exhibit a positive correlation with the viral load of EVs HCVRNA, suggesting a direct association between the expression of EVs miRNA-155 and HCV replication. Furthermore, the expression of EVs miRNA-122 is significantly higher in the genotype 1b SVR infection group compared to the genotype 2a and genotype 6a infection groups, indicating a potential association between elevated expression of miRNA-122 in EVs and a favorable treatment outcome (58).

It is noteworthy that despite the promising diagnostic potential of EVs, their utilization as biomarkers for infectious liver diseases remains an area of active research. Standardized methods for the isolation, characterization, and analysis of EVs need to be established to ensure reliability, accuracy, and reproducibility. Furthermore, the identification of specific EVs biomarkers and the validation of their clinical utility still pose challenges in this field.

## 9 Application of extracellular vesicles as therapeutic agents or carriers

Abundant research indicates that factors released by mesenchymal stem cells (MSCs) can induce liver repair and ameliorate systemic inflammation through their paracrine effects. This paracrine action is not only based on the secretion of cytokines and growth factors but also relies on EVs. Studies have demonstrated that EVs derived from MSCs can inhibit hepatocyte apoptosis, support hepatic function (59), promote angiogenesis (60) and enhance hepatocyte proliferation (61), Furthermore, they play a role in promoting liver health by reducing inflammatory responses, preventing immune cell infiltration, or suppressing the release of inflammatory cytokines (59). Moreover, research based on animal models suggests that EVs may represent a novel and effective non-cellular therapeutic agent, serving as an alternative to cell therapy for patients with liver diseases (62). Therefore, therapeutic interventions mediated by MSC-derived extracellular vesicles (MSC-EVs) hold the promise of offering an innovative, non-cellular, non-invasive, low immunogenic, and non-toxic alternative strategy for liver treatment. Additionally, they provide crucial new insights into mechanisms for hepatic cell repair functions (63).

Currently, research on engineered MSC-EVs as drug delivery vehicles and/or targeting systems for liver diseases is in its early stages (64). EVs can activate the immune functions of the human body to respond to infections, exhibiting a bidirectional role in both viral dissemination and resistance to viral responses. Therefore, EVs, as candidates for antiviral agents or vaccines, have tremendous potential for treating viral hepatitis (15). EVs can also serve as biological, targeted, relatively stable, and promising carriers for delivering therapeutic drugs or agents against HCV infection and HCV-mediated liver diseases (65).

Beyond native MSC-derived EVs, engineered EVs are emerging as promising delivery systems for antiviral and antifibrotic therapy. EVs can be modified by surface engineering to improve liver targeting or loaded with therapeutic cargos such as siRNAs, miRNA mimics, anti-miRNA oligonucleotides, small-molecule antivirals, proteins, or genome-editing components. In HBV infection, potential cargos may include siRNAs targeting HBV transcripts, molecules that enhance antiviral interferon signaling, or agents that reduce immune exhaustion. In HCV-related disease, engineered EVs may be used to deliver miR-122 inhibitors or antifibrotic RNAs to hepatocytes and hepatic stellate cells. Compared with synthetic nanoparticles, EVs offer advantages such as biocompatibility, low immunogenicity, membrane stability, and natural tropism toward specific tissues. Nevertheless, several barriers remain before clinical translation, including scalable production, cargo-loading efficiency, batch consistency, biodistribution control, long-term safety, and avoidance of unintended viral or oncogenic cargo transfer (66). Recent reviews also emphasize that engineered EVs may become useful therapeutic tools in liver diseases, but current evidence remains largely preclinical (67, 68).

## 10 Current challenges and limitations

Despite rapid progress, several limitations restrict the clinical application of EVs in HBV and HCV research. First, EV preparations are highly heterogeneous and may contain exosomes, microvesicles, apoptotic bodies, lipoproteins, protein aggregates, subviral particles, and complete virions. This is particularly problematic in viral hepatitis because HBV subviral particles and HCV lipoviroparticles may overlap with EVs in physical properties. Second, EV isolation methods, including ultracentrifugation, size-exclusion chromatography, precipitation, immunoaffinity capture, and microfluidic approaches, differ in yield, purity, cost, and scalability. These differences can lead to inconsistent biomarker results across studies. Third, many findings remain based on cell lines, animal models, or small clinical cohorts, and large prospective validation studies are still lacking. Fourth, EVs may exert both protective and pathogenic effects, raising safety concerns for therapeutic use. Therefore, future studies should follow standardized reporting guidelines, include appropriate EV-negative and virion-depleted controls, and combine molecular profiling with functional assays to distinguish association from causality.

## 11 Prospects

The relationship between infectious hepatitis and EVs constitutes a promising area of research that could have profound implications for future developments. Serving as a crucial mode of intercellular communication, EVs participate in the spread and infection processes of hepatitis viruses by carrying viral particles or virus-related bioactive substances. The content of EVs, including proteins, nucleic acids, and lipids, is also involved in various processes such as viral dissemination, immune responses, and inflammatory reactions, influencing the progression of diseases and clinical manifestations following hepatitis virus infection. In the future, a deeper understanding of the specific mechanisms and impacts of EVs is expected to provide a comprehensive perspective for the innovation of therapeutic strategies, discovery of biomarkers, personalized treatment, vaccine development, and the interdisciplinary exploration of virology and immunology in the context of infectious hepatitis research.

Future studies should move from descriptive cargo profiling toward mechanism-driven and clinically validated research. Single-EV analysis, spatial omics, high-resolution flow cytometry, digital PCR, and multi-omics integration may help distinguish hepatocyte-derived, immune-cell-derived, and tumor-derived EV subpopulations. Liver organoids, humanized mouse models, and patient-derived samples could further clarify how EVs regulate viral persistence and liver disease progression in physiologically relevant systems. In clinical translation, longitudinal studies are needed to determine whether EV signatures can predict antiviral response, fibrosis progression, HCC recurrence, or post-SVR complications better than existing markers. Ultimately, combining EV-based liquid biopsy with conventional virological, biochemical, imaging, and histological indicators may provide a more precise framework for personalized management of HBV- and HCV-related liver diseases.
